# Supplementary material for: Possible sarcopenia and depression among middle-aged and older adults in China: A 9-year longitudinal survey
Source: PLoS One. 2025 Apr 3;20(4):e0318666. doi: 10.1371/journal.pone.0318666 (PMC11967972; doi:10.1371/journal.pone.0318666)
Supplement: S1 Table — (DOCX) [file pone.0318666.s001.docx]

**S1 Table. Cross-sectional association of PS and its components with depression at baseline**

|  | ***β*** | ***SE*** | ***Wild-χ^2^*** | ***P*** | ***OR（95%CI)*** |
| --- | --- | --- | --- | --- | --- |
| **Association of PS and depression** |  |  |  |  |  |
| PS | 0.387 | 0.050 | 59.931 | <0.001 | 1.47 (1.34,1.63) |
| Age,years |  |  |  |  |  |
| 45-49 | 1.000 |  |  |  |  |
| 50-59 | -0.068 | 0.067 | 1.025 | 0.311 | 0.94 (0.82,1.07) |
| 60-69 | -0.136 | 0.072 | 3.576 | 0.059 | 0.87 (0.76,1.01) |
| ≥70 | -0.327 | 0.089 | 13.510 | <0.001 | 0.72 (0.61,0.86) |
| Rgender (Male Vs. Female) | 0.369 | 0.048 | 59.412 | <0.001 | 1.45 (1.32,1.59) |
| Education level |  |  |  |  |  |
| Primary school and below | 1.000 |  |  |  |  |
| Middle school | -0.253 | 0.061 | 17.040 | <0.001 | 0.78 (0.69,0.88) |
| High School or above | -0.621 | 0.088 | 49.629 | <0.001 | 0.54 (0.45,0.64) |
| Married (Vs. Other) | 0.467 | 0.072 | 42.646 | <0.001 | 1.60 (1.39,1.84) |
| Urban residence (Vs. Rural) | 0.346 | 0.920 | 14.172 | <0.001 | 1.41 (1.18,1.69) |
| Sleep duration ,hours |  |  |  |  |  |
| ≤6 | 1.000 |  |  |  |  |
| 6 to 8 | -0.731 | 0.054 | 183.812 | <0.001 | 0.48 (0.43,0.54) |
| >8 | -0.918 | 0.059 | 240.654 | <0.001 | 0.40 (0.36,0.45) |
| With afternoon napping (Vs. no) | -0.096 | 0.046 | 4.327 | 0.038 | 0.91 (0.83,0.99) |
| BMI,kg/m2 |  |  |  |  |  |
| <24.0 | 1.000 |  |  |  |  |
| 24.0 to 27.9 | -0.211 | 0.053 | 15.534 | <0.001 | 0.81 (0.73,0.90) |
| ≥28 | -0.406 | 0.078 | 27.059 | <0.001 | 0.67 (0.57,0.78) |
| Hypertension | -0.107 | 0.048 | 4.894 | 0.027 | 0.90 (0.82,0.99) |
| Stroke | 0.332 | 0.072 | 21.446 | <0.001 | 1.39 (1.21,1.60) |
| Heart disease | 0.463 | 0.162 | 8.205 | 0.004 | 1.59 (1.16,2.18) |
| Arthritis or rheumatism | 0.547 | 0.048 | 132.764 | <0.001 | 1.73 (1.58,1.90) |
| Health self-assessment |  |  |  |  |  |
| Good | 1 |  |  |  |  |
| Fair | 0.654 | 0.05 | 170.998 | <0.001 | 1.92 (1.74,2.12) |
| Poor | 1.336 | 0.067 | 394.879 | <0.001 | 3.81 (3.34,4.34) |
| **Association of LMS and depression** |  |  |  |  |  |
| LMS | 0.377 | 0.082 | 21.221 | <0.001 | 1.46 (1.24,1.71) |
| Age,years |  |  |  |  |  |
| 45-49 | 1.000 |  |  |  |  |
| 50-59 | -0.044 | 0.067 | 0.438 | 0.508 | 0.96 (0.84,1.09) |
| 60-69 | -0.094 | 0.072 | 1.719 | 0.190 | 0.91 (0.79,1.05) |
| ≥70 | -0.241 | 0.089 | 7.405 | 0.007 | 0.79 (0.66,0.94) |
| Rgender (Male Vs. Female) | 0.410 | 0.065 | 39.848 | <0.001 | 1.51 (1.33,1.71) |
| Education level |  |  |  |  |  |
| Primary school and below | 1.000 |  |  |  |  |
| Middle school | -0.294 | 0.061 | 18.627 | <0.001 | 0.77 (0.68,0.87) |
| High School or above | -0.638 | 0.088 | 52.521 | <0.001 | 0.53 (0.45,0.63) |
| Married (Vs. Other) | 0.464 | 0.714 | 42.245 | <0.001 | 1.59 (1.38,1.83) |
| Urban residence (Vs. Rural) | 0.359 | 0.092 | 15.206 | <0.001 | 1.43 (1.20,1.71) |
| Sleep duration ,hours |  |  |  |  |  |
| ≤6 | 1.000 |  |  |  |  |
| 6 to 8 | -0.729 | 0.054 | 183.797 | <0.001 | 0.48 (0.43,0.54) |
| >8 | -0.910 | 0.006 | 237.895 | <0.001 | 0.40 (0.36,0.45) |
| With afternoon napping (Vs. no) | -0.103 | 0.046 | 4.975 | 0.026 | 0.90 (0.82,0.99) |
| BMI,kg/m2 |  |  |  |  |  |
| <24.0 | 1.000 |  |  |  |  |
| 24.0 to 27.9 | -0.192 | 0.054 | 12.882 | <0.001 | 0.83 (0.74,0.92) |
| ≥28 | -0.374 | 0.078 | 23.083 | <0.001 | 0.69 (0.59,0.80) |
| Hypertension | -0.095 | 0.048 | 3.913 | 0.048 | 0.91 (0.83,1.00) |
| Stroke | 0.359 | 0.072 | 25.216 | <0.001 | 1.43 (1.25,1.65) |
| Heart disease | 0.510 | 0.161 | 10.067 | 0.002 | 1.67 (1.22,2.28) |
| Arthritis or rheumatism | 0.539 | 0.047 | 129.411 | <0.001 | 1.71 (1.56,1.88) |
| Health self-assessment |  |  |  |  |  |
| Good | 1 |  |  |  |  |
| Fair | 0.661 | 0.05 | 175.193 | <0.001 | 1.94 (1.76 (2.14) |
| Poor | 1.367 | 0.067 | 412.714 | <0.001 | 3.92 (3.44,4.47) |
| **Association of LPP and depression** |  |  |  |  |  |
| LPP | 0.374 | 0.511 | 53.739 | <0.001 | 1.45 (1.31,1.61) |
| Age,years |  |  |  |  |  |
| 45-49 | 1.000 |  |  |  |  |
| 50-59 | -0.061 | 0.067 | 0.823 | 0.364 | 0.94 (0.83,1.07) |
| 60-69 | -0.116 | 0.072 | 2.599 | 0.107 | 0.89 (0.77,1.03) |
| ≥70 | -0.281 | 0.089 | 10.046 | 0.002 | 0.76 (0.64,0.90) |
| Rgender (Male Vs. Female) | 0.372 | 0.065 | 32.642 | <0.001 | 1.45 (1.28,1.65) |
| Education level |  |  |  |  |  |
| Primary school and below | 1.000 |  |  |  |  |
| Middle school | -0.259 | 0.061 | 17.818 | <0.001 | 0.77 (0.69,0.87) |
| High School or above | -0.621 | 0.088 | 49.588 | <0.001 | 0.54 (0.45,0.64) |
| Married (Vs. Other) | 0.472 | 0.072 | 43.558 | <0.001 | 1.60 (1.39,1.85) |
| Urban residence (Vs. Rural) | 0.350 | 0.092 | 14.442 | <0.001 | 1.42 (1.19,1.70) |
| Sleep duration ,hours |  |  |  |  |  |
| ≤6 | 1.000 |  |  |  |  |
| 6 to 8 | -0.732 | 0.054 | 184.307 | <0.001 | 0.40 (0.36,0.45) |
| >8 | -0.919 | 0.059 | 241.491 | <0.001 | 0.48 (0.43,0.54) |
| With afternoon napping (Vs. no) | -0.095 | 0.046 | 4.226 | 0.040 | 0.91 (0.83,1.00) |
| BMI,kg/m2 |  |  |  |  |  |
| <24.0 | 1.000 |  |  |  |  |
| 24.0 to 27.9 | -0.207 | 0.054 | 14.921 | <0.001 | 0.81 (0.73,0.90) |
| ≥28 | -0.400 | 0.078 | 26.024 | <0.001 | 0.67 (0.58,0.78) |
| Hypertension | -0.108 | 0.048 | 5.019 | 0.025 | 0.90 (0.82,0.99) |
| Stroke | 0.340 | 0.072 | 22.462 | <0.001 | 1.41 (1.22,1.62) |
| Heart disease | 0.473 | 0.162 | 8.507 | 0.004 | 1.60 (1.17,2.20) |
| Arthritis or rheumatism | 0.547 | 0.048 | 132.690 | <0.001 | 1.73 (1.58,1.90) |
| Health self-assessment |  |  |  |  |  |
| Good | 1 |  |  |  |  |
| Fair | 0.658 | 0.05 | 173.26 | <0.001 | 1.93 (1.75,2.13) |
| Poor | 1.352 | 0.067 | 402.854 | <0.001 | 3.86 (3.39,4.41) |

Age gender, education, marital status and area of residence, smoking, alcohol consumption, sleep duration and afternoon napping, BMI and number of chronic diseases were included.

*OR*, odds ratio; *CI*, confidence interval; PS, possible sarcopenia; LMS, low muscle strength; LPP, low physical performance.
