# Supplementary material for: Possible sarcopenia and depression among middle-aged and older adults in China: A 9-year longitudinal survey
Source: PLoS One. 2025 Apr 3;20(4):e0318666. doi: 10.1371/journal.pone.0318666 (PMC11967972; doi:10.1371/journal.pone.0318666)
Supplement: S2 Table — (DOCX) [file pone.0318666.s002.docx]

**S2 Table. Longitudinal association of PS and its components with depression**

|  | ***β*** | ***SE*** | ***Wild-χ^2^*** | ***P*** | ***OR（95%CI)*** |
| --- | --- | --- | --- | --- | --- |
| **Association of PS and depression** |  |  |  |  |  |
| PS | 0.108 | 0.041 | 6.861 | 0.009 | 1.11 (1.03,1.21) |
| Age,years |  |  |  |  |  |
| 45-49 | 1.000 |  |  |  |  |
| 50-59 | 0.042 | 0.051 | 0.682 | 0.409 | 1.04 (0.91,1.15) |
| 60-69 | -0.022 | 0.055 | 0.161 | 0.688 | 0.98 (0.88,1.09) |
| ≥70 | -0.223 | 0.072 | 9.487 | 0.002 | 0.80 (0.70,0.92) |
| Rgender (Male Vs. Female) | 0.315 | 0.037 | 71.042 | <0.001 | 1.37 (1.27,1.47) |
| Education level |  |  |  |  |  |
| Primary school and below | 1.000 |  |  |  |  |
| Middle school | -0.238 | 0.048 | 24.794 | <0.001 | 0.79 (0.72,0.87) |
| High School or above | -0.463 | 0.066 | 49.505 | <0.001 | 0.63 (0.55,0.72) |
| Married (Vs. Other) | 0.139 | 0.063 | 4.813 | 0.028 | 1.15 (1.02,1.30) |
| Urban residence (Vs. Rural) | 0.478 | 0.076 | 39.785 | <0.001 | 1.61 (1.39,1.87) |
| Sleep duration ,hours |  |  |  |  |  |
| ≤6 | 1.000 |  |  |  |  |
| 6 to 8 | -0.208 | 0.046 | 20.814 | <0.001 | 0.81 (0.74,0.89) |
| >8 | -0.256 | 0.048 | 27.902 | <0.001 | 0.77 (0.70,0.85) |
| Arthritis or rheumatism | 0.134 | 0.039 | 11.490 | 0.001 | 1.14 (1.06,1.24) |
| Health self-assessment |  |  |  |  |  |
| Good | 1.000 |  |  |  |  |
| Fair | 0.237 | 0.039 | 37.571 | <0.001 | 1.26 (1.17,1.36) |
| Poor | 0.371 | 0.061 | 36.470 | <0.001 | 1.45 (1.29,1.64) |
| **Association of LMS and depression** |  |  |  |  |  |
| LMS | 0.157 | 0.074 | 4.466 | 0.035 | 1.17 (1.01,1.35) |
| Age,years |  |  |  |  |  |
| 45-49 | 1.000 |  |  |  |  |
| 50-59 | 0.049 | 0.051 | 0.920 | 0.337 | 1.05 (0.95,1.16) |
| 60-69 | -0.016 | 0.055 | 0.080 | 0.776 | 0.99 (0.88,1.10) |
| ≥70 | -0.212 | 0.072 | 8.636 | 0.003 | 0.81 (0.70,0.93) |
| Rgender (Male Vs. Female) | 0.324 | 0.037 | 75.913 | <0.001 | 1.38 (1.29,1.49) |
| Education level |  |  |  |  |  |
| Primary school and below | 1.000 |  |  |  |  |
| Middle school | -0.241 | 0.048 | 25.428 | <0.001 | 0.79 (0.72,0.86) |
| High School or above | -0.467 | 0.066 | 50.504 | <0.001 | 0.63 (0.55,0.71) |
| Married (Vs. Other) | 0.136 | 0.063 | 4.607 | 0.032 | 1.145 (1.01,1.30) |
| Urban residence (Vs. Rural) | 0.478 | 0.076 | 39.836 | <0.001 | 1.61 (1.39,1.87) |
| Sleep duration ,hours |  |  |  |  |  |
| ≤6 | 1.000 |  |  |  |  |
| 6 to 8 | -0.210 | 0.046 | 21.341 | <0.001 | 0.81 (0.74,0.89) |
| >8 | -0.255 | 0.048 | 27.723 | <0.001 | 0.78 (0.71,0.85) |
| Arthritis or rheumatism | 0.131 | 0.039 | 11.036 | 0.001 | 1.14 (1.06,1.23) |
| Health self-assessment |  |  |  |  |  |
| Good | 1 |  |  |  |  |
| Fair | 0.239 | 0.039 | 38.258 | <0.001 | 1.27 (1.18,1.38) |
| Poor | 0.37 | 0.062 | 36.058 | <0.001 | 1.45 (1.28,1.63) |
| **Association of LPP and depression** |  |  |  |  |  |
| LPP | 0.092 | 0.042 | 4.731 | 0.030 | 1.10 (1.01,1.19) |
| Age,years |  |  |  |  |  |
| 45-49 | 1.000 |  |  |  |  |
| 50-59 | 0.052 | 0.051 | 1.043 | 0.307 | 1.05 (0.95,1.16) |
| 60-69 | 0.000 | 0.055 | 0.000 | 0.996 | 1.00 (0.90,1.11) |
| ≥70 | -0.197 | 0.072 | 7.580 | 0.006 | 0.82 (0.71,0.95) |
| Rgender (Male Vs. Female) | 0.325 | 0.037 | 75.880 | <0.001 | 1.38 (1.29,1.49) |
| Education level |  |  |  |  |  |
| Primary school and below | 1.000 |  |  |  |  |
| Middle school | -0.253 | 0.048 | 28.046 | <0.001 | 0.78 (0.71,0.85) |
| High School or above | -0.462 | 0.066 | 49.638 | <0.001 | 0.63 (0.55,0.72) |
| Married (Vs. Other) | 0.134 | 0.063 | 4.534 | 0.033 | 1.14 (1.01,1.29) |
| Urban residence (Vs. Rural) | 0.483 | 0.076 | 40.901 | <0.001 | 1.62 (1.40,1.88) |
| Sleep duration ,hours |  |  |  |  |  |
| ≤6 | 1.000 |  |  |  |  |
| 6 to 8 | -0.218 | 0.045 | 23.113 | <0.001 | 0.80 (0.74,0.88) |
| >8 | -0.268 | 0.048 | 30.790 | <0.001 | 0.77 (0.70,0.84) |
| Arthritis or rheumatism | 0.163 | 0.039 | 17.359 | <0.001 | 1.18 (1.09,1.27) |
| Health self-assessment |  |  |  |  |  |
| Good | 1.000 |  |  |  |  |
| Fair | 0.240 | 0.038 | 38.913 | <0.001 | 1.27 (1.18,1.37) |
| Poor | 0.373 | 0.061 | 37.628 | <0.001 | 1.45 (1.29,1.64) |

Age gender, education, marital status and area of residence, smoking, alcohol consumption, sleep duration and afternoon napping, BMI and number of chronic diseases were included.

*OR*, odds ratio; *CI*, confidence interval; PS, possible sarcopenia; LMS, low muscle strength; LPP, low physical performance.
